# Systematic framework for performance evaluation of exoskeleton actuators

**DOI:** 10.1017/wtc.2020.5

**Published:** 2020-10-01

**Authors:** Christian Di Natali, Stefano Toxiri, Stefanos Ioakeimidis, Darwin G. Caldwell, Jesús Ortiz

**Affiliations:** Department of Advanced Robotics, Istituto Italiano di Tecnologia, Genoa, Italy

**Keywords:** Task analysis, Actuators, Torque control, Exoskeletons, Dynamics, Test-bench

## Abstract

Wearable devices, such as exoskeletons, are becoming increasingly common and are being used mainly for improving motility and daily life autonomy, rehabilitation purposes, and as industrial aids. There are many variables that must be optimized to create an efficient, smoothly operating device. The selection of a suitable actuator is one of these variables, and the actuators are usually sized after studying the kinematic and dynamic characteristics of the target task, combining information from motion tracking, inverse dynamics, and force plates. While this may be a good method for approximate sizing of actuators, a more detailed approach is necessary to fully understand actuator performance, control algorithms or sensing strategies, and their impact on weight, dynamic performance, energy consumption, complexity, and cost. This work describes a learning-based evaluation method to provide this more detailed analysis of an actuation system for our *XoTrunk* exoskeleton. The study includes: (a) a real-world experimental setup to gather kinematics and dynamics data; (b) simulation of the actuation system focusing on motor performance and control strategy; (c) experimental validation of the simulation; and (d) testing in real scenarios. This study creates a systematic framework to analyze actuator performance and control algorithms to improve operation in the real scenario by replicating the kinematics and dynamics of the human–robot interaction. Implementation of this approach shows substantial improvement in the task-related performance when applied on a back-support exoskeleton during a walking task.

## 1 Introduction

### 1.1 Exoskeletons and Applications

The past few years have seen rapidly growing interest in exoskeletons and their applications (Ferris and Schlink, 2017; Young and Ferris, 2017). These are wearable devices that support physical activities by working in synchrony with one or more joints of the musculoskeletal structure. The most common field where exoskeletons are applied is physical/motor rehabilitation using systems such as Lokomat (Jezernik et al., 2003) and LOPES (Veneman et al., 2007), both of which are static/fixed structures. In contrast, mobile exoskeletons have the potential to be used outside clinical settings to restore some degree of motility to people with pathologies causing severe loss of mobility. For example, bilateral ankle-knee-hip assistive devices aim to enable paraplegics to stand upright, walk, and climb stairs (Farris et al., 2011; Murray et al., 2014). Simpler devices targeted at assisting people with moderate to low impairments, such as the elderly, usually assist a single or double joint (Kong and Jeon, 2006; Ikehara et al., 2011).

Beyond rehabilitation, a rapidly developing application field is industry, (Sugar et al., 2018; Toxiri et al., 2019), where the intelligence of human operators and the strength, precision, and endurance of industrial robots are combined (De Looze et al., 2016). The main objective of industrial exoskeletons is the prevention of work-related musculoskeletal disorders (MSDs). The most common health problems are pain located in the back and shoulders, overall fatigue, and resulting stress. Prevention of these injuries is essential to decrease the number of incidences, their costs and the resultant burden on society.

More recently, a new paradigm of exoskeletons using soft wearable structures has been proposed. These systems, often called exosuits (Cappello et al., 2016; Awad et al., 2017; Jin et al., 2017; Schmidt et al., 2017; Di Natali et al., 2019), are much lighter than conventional designs and actuate or restrain the joints using soft or hybrid structures and active tendon driven actuators.

### 1.2 Challenges in Sizing Actuators for Exoskeletons

How best to integrate an actuator’s performances with the user’s requirements, such as comfort and effectiveness of the assistance, is a common question for exoskeleton developers (Toxiri et al. (2017), Toxiri et al. (2018b), Calanca et al. (2020)). Addressing all the often-competing issues becomes a tradeoff between the device performance and the mitigation of associated drawbacks. For example, trying to increase the level of assistance may result in over dimensioned actuators making the system less reactive, slow, and heavy.

Actuators on the vast majority of exoskeletons use indirect drives based on electrical brushless DC (BLDC) motors, reduction gears, more recently fixed and variable compliant systems, etc. (Laffranchi et al., 2011; Wang et al., 2011; Beckerle et al., 2017), but this creates increasingly complex drive structure. Usually, these actuators are controlled by velocity and/or torque sensors to fully or partially provide support for the coupled human user. To properly size the actuators, the dynamic characteristics of the human joint of interest should be analyzed within the context of the target task. Information coming from the motion tracking system and force plates is typically used in combination with inverse dynamics biomechanical models in order to estimate the torques at the target joints (Winter, 1991; Di Gironimo et al., 2012). While this is a good approach for approximate sizing of the actuators, a more detailed approach is necessary to optimize the design and ensure safety in the actuator performance, control and/or, sensing strategy. In fact, a possible first approximation is that the kinematics of the exoskeleton correspond to those of the human body. However, this approximation may not always be accurate enough, since the exoskeleton actuators will probably not be spatially collocated with the wearer’s muscles or joints. In fact, no exoskeleton can achieve perfect kinematics compatibility with the corresponding human joints (Näf et al., 2018); therefore, a degree of simplification must be tolerated. Thus, both anthropomorphic and nonanthropomorphic exoskeletons must have kinematic misalignment compensation strategies. Controlled design for wearable robots must also take into consideration the human interaction, which may radically affect control performance. From a control point of view, wearers can be considered as a disturbance; therefore, interaction forces between the device, the user, and eventually, the environment, cannot be taken into account a priori, and if perceived, the controller should be able to strongly reject these disturbances (Tucker et al., 2015). The complexity of the whole system does not allow for approximate controllers that would generate uncomfortable effects on the wearer. Thus, a different approach to dimensioning of the exoskeleton’s control that also takes into consideration the human–robot interaction needs to be adopted. To prevent over- or undersizing of the actuators, we propose to base the mechatronic solutions and control strategies on task-specific kinematics data derived from the exoskeleton joint, instead of exploiting the kinematics of the corresponding human joint.

### 1.3 Presented Framework

To overcome the drawbacks of overdimensioned actuators on exoskeletons, this work introduces a learning-based systematic approach to the sizing and evaluation of actuation solutions. The rationale is to find a close match between actuator performance and task requirements. This will lead to the most efficient use of the actuator. In this paper, we apply this approach to a back-support exoskeleton used to assist workers performing manual handling tasks.

**Figure 1.**
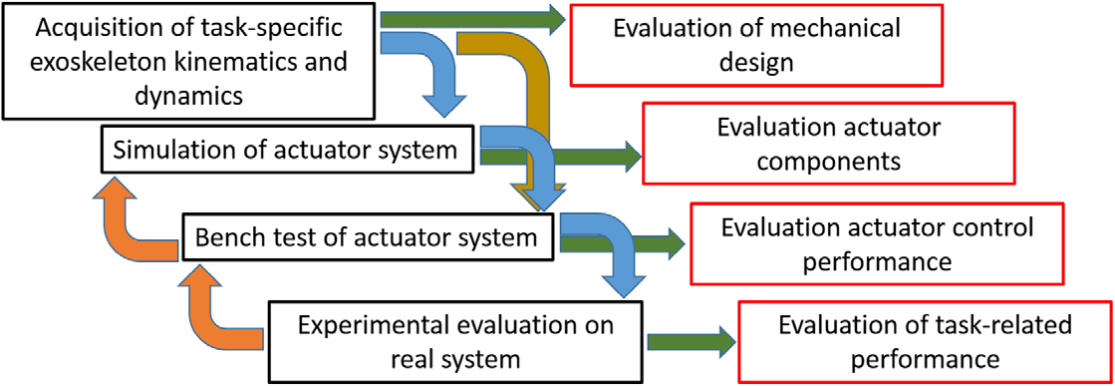
Flow diagram of the systematic approach to improve overall exoskeleton performance. Black boxes represent the four main steps of the systematic analysis. Possible outcomes are shown in red.

As illustrated in Figure 1, this approach consists of a sequence of steps, each of them tackling a specific challenge in sizing of the actuators. This iterative approach used here is common in the development of state-of-the-art systems (e.g., Shore et al., 2018) and aims to improve the design choices leading to the selection of the actuators and controllers and thereby improving the overall device performance. Each step may be iterated more than once to improve the final results. First, (a) task-specific data are acquired directly from the exoskeleton, extracting the kinematic and dynamic information that will be used for the selection of the actuator. Use of this user-gathered data provide a more precise analysis of the kinematics, although there is slightly more effort needed to perform the experimental/data gathering evaluation. This technique creates a much better mapping than can be achieved in conventional approaches that simply use the closest corresponding human joint to approximate the actuator behavior. Second, (b) task-specific data are used to support component selection by developing a computer simulation of the whole system comprehensive of actuator and human interaction. Computer simulation of the actuation system helps to analyze the behavior of the actuator and controller during the interaction with the wearer. This interaction is modeled as an auxiliary disturbance motor. This simulation/model is validated in the third step (c) which implements the same system in a physical test-bench setup. Last, (d) the performance of the selected actuators and controllers are tested on the exoskeleton in a series of real scenarios.

The development of exoskeletons is a complicated practice due to the nature of interaction with the human being. Such interaction, indeed, is a biomechanical interaction where both actors (i.e., human and exoskeleton) apply controlled forces to the limbs. The key contributions of this work are: the use of task-specific data gathered directly from the device (and not from estimates of the user’s musculoskeletal structures), kinematic analysis of a wide task selection, identification of the worst scenario from kinematic and dynamic standpoint, and assessment of the model simulation to validate its use as a development tool. This work thus aims to present this system development systematic approach, which takes strongly in consideration the human–robot interaction, by developing a computer simulator, and a test-bench that replicates kinematics and dynamics of the human–robot interaction while carrying out specific tasks.

The paper is structured as follows: “XoTrunk Exoskeleton Prototype” presents the exoskeleton prototype and the task selected as the case study. “Systematic Framework” describes the systematic framework in detail, breaking it into subsections according to the steps outlines above. Finally, “Conclusions” draws conclusions and suggests future developments.

## 2 XoTrunk Exoskeleton Prototype

**Figure 2.**
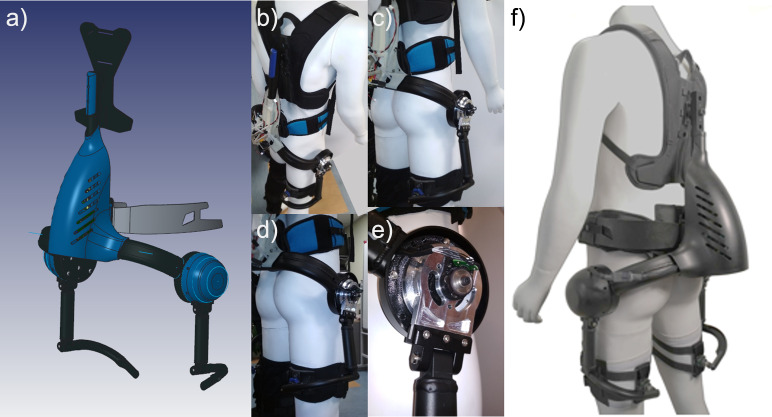
(a) Rendering of the *XoTrunk* prototype structure and body attachments. (b–e) Pictures of the prototype B without actuators *XoTrunk* with embedded encoders and electronics. (f) Pictures of *XoTrunk* (prototype A) mounting the actuators.

The *XoTrunk* prototype is a torque-controlled back-support exoskeleton (see Figure 2) developed in collaboration with INAIL (Italian Workers’ Compensation Authority) at the *XoLab* at Istituto Italiano di Tecnologia (IIT). The exoskeleton is designed to reduce spinal loads during manual handling tasks (Toxiri et al., 2015; Toxiri et al., 2018a). *XoTrunk* (Figure 2f) is composed of a tubular aluminum frame, with attachments on the torso consisting of backpack-like shoulder and waist straps and thigh bands. The hip actuators (one on each side of the body) generate torque (up to 30 Nm continuous and 70 Nm peak) between the torso and corresponding thigh links by pivoting on the hip/waist. The assistive torque is provided only in the sagittal plane, and the rotation axis of the actuator is approximately aligned with the hip flexion–extension axis. Overall, the prototype weighs approximately 6 kg. Each actuator assembly uses a BLDC motor EC60-flat, 100 W and 24 V supply (Maxon Motor AG, Switzerland), a Harmonic Drive SHD20 with 1:100 reduction (HD System, Inc), and a torque sensor TS110-A (ME-Messsysteme GmbH, Germany) with 100 Nm full scale. The exoskeleton configuration with actuators is identified as prototype A.

Figure 2a shows the rendering of the *XoTrunk* exoskeleton (prototype A), while Figure 2b–e show the prototype B. This prototype B exoskeleton does not have any actuators mounted and is used in the experimental trials in “Systematic Framework” (more details in that section).

**Figure 3.**
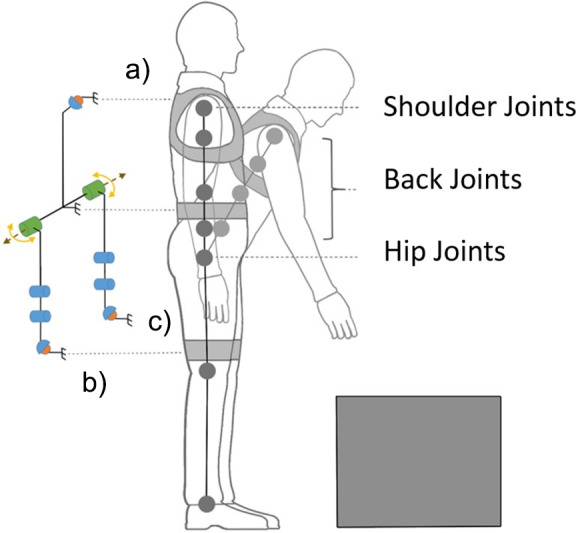
Kinematic structure of the humanoid model together with the *XoTrunk* schematic.

Figure 3 shows the schematic of the *XoTrunk* kinematics and body attachments on a simplified human model (Toxiri et al., 2015). The exoskeleton has two actuated joints represented in green. Spherical joints for misalignment compensation are located at the shoulders (A) and thighs (B and C). Two pairs of rotational joints on each side, connecting the actuators to the leg attachments (B or C), allow hip adduction/abduction.

## 3 Systematic Framework

This section describes the four steps of the framework, as previously introduced and illustrated in Figure 1. First, the realistic task-specific data (three different tasks, each presented in two formats) are acquired, analyzed, and associated with the actuator kinematics (“Actuator Kinematics From Task-Specific Realistic Data”). Second, two of the most representative tasks are used in a model-based computer simulation assessment that guides the component selection (“Simulation of Actuation Performance”). The third step implements the simulated system in a physical benchtop setup (“Experimental Validation of Actuator Performance”). The task selected for implementation corresponds to the most demanding scenario in terms of high kinematic variability. Last, the resulting performance is observed in a real scenario, implementing the selected actuator, and controls on the exoskeleton in a realistic task of interest (“Real-Scenario Evaluation of Actuation Performance”).

### 3.1 Actuator Kinematics from Task-Specific Realistic Data

The kinematics of an exoskeleton actuator cannot be realistically approximated by those of the corresponding human joint, at least not well-enough for the purpose of sizing of the actuator. For example, due to kinematic mismatch (as will be shown later), the exoskeleton actuator may need to accelerate faster than the corresponding human joint, with implications on its required performance. To overcome this limitation, the framework developed in this paper attempts to capture more closely the kinematics that the actuator will experience during operational use. In the following section, we illustrate the kinematic mismatch on the exoskeleton and describe an experiment to quantify the mismatch.

#### 3.1.1 Kinematic considerations

Figure 3 shows the schematics of the exoskeleton (Toxiri et al., 2015) and the human model. The human spine is kinematically very complex with even simplified models of the lumbar and thoracic spine having 17 rigid bodies and 51 degrees of freedom (DOF) (Simonidis et al., 2007). Moreover such complexity, a simplification on the kinematic representation of the spine is widely adopted and accepted in the scientific community. The spinal column could be modeled as a series of interdependent joints with three degrees of rotational freedom (Monheit and Badler, 1990).

The exoskeleton’s kinematic chain is composed of a rigid frame attached to user’s torso using shoulder straps (point A in Figure 3) and a waist belt. At the shoulder point, a spherical joint is used to compensate for eventual kinematic incompatibilities (Näf et al., 2018). Two motors, on the sides, are attached to the frame (represented in green in Figure 3). They rotate the exoskeleton’s leg links connected to the user’s thigh (point B and C in Figure 3). From the motor to each of the leg attachments, a R-R-S (rotational-rotational-spherical) self-alignment mechanism, composed by passive joints, is employed to compensate for the migration of the motor’s instantaneous center of rotation with respect to the hip one. The two parallel kinematic chains, the human and the exoskeleton, between the attaching points A and B (or C), and projected on the sagittal plane (shown in Figure 3), show one redundant DOF. The exoskeleton has three rotational DOFs (projection of two spherical joint used for kinematic compensation and the actuated joint at the hip level), and the human kinematic model, comprehensive of spinal column–simplified model and hip joint, has four rotational DOFs. Thus, from a kinematic analysis point of view, it is evident that a nonanthropomorphic exoskeleton cannot fully replicate the kinematics of the human joints. Therefore, human and exoskeleton kinematics are expected to show differences during the motion in terms of angle position, speed, and acceleration.

#### 3.1.2 Experimental setup

Experiments were carried out using B prototype of the *XoTrunk* exoskeleton. Prototype B consists of the unactuated version of *XoTrunk*, where the structure, joints, and body attachments together with the sensing strategy are the same as in its original version (prototype A) displayed in Figure 2f, but prototype A has two actuators, two torque sensors, and electronics to enable assistive control. For this first study in prototype B, these components have been removed in order to avoid any constraints due to the friction and inertia of the actuation system. Thus, minimal external forces, friction, or mechanical encumbrance affect the system or human dynamics. This exoskeleton configuration embeds the main electronics (Raspberry Pi 3, The Raspberry Pi Foundation, UK), and a 9-DOF IMU (BNO055, Bosch, Gerlingen, Germany) on the rear of the exoskeleton, with two absolute magnetic encoders, one for each side. These ic-MU encoders (IC-HAUS, Bodenheim, Germany) measure the exoskeleton’s joint displacements during specific user tasks and have a resolution of 16 bits (equivalent to 0.0055^o^). The system runs at 2 kHz.

#### 3.1.3 Experimental test protocol

The experimental trials were conducted in a controlled laboratory environment and included walking and lifting tasks conducted by one healthy subject (male, 30 years old, 1.7 m tall, 70 kg). During the test, the participant’s full-body kinematics and the exoskeleton’s on-board sensors were recorded. An Xsens wearable motion tracking system was used (MTw Awinda 3D Wireless Motion Tracker, Xsens Technologies B.V. Enschede, the Netherlands). These tests are based on the experimental protocol approved by the Ethics Committee of Liguria, Italy (protocol number: 001/2019). The subject performed each of the tasks wearing the prototype B of the exoskeleton described in “XoTrunk Exoskeleton Prototype.” The walking tests were conducted on a treadmill at two constant speeds of 2.5 km/hr and 5 km/hr (0.7 m/s and 1.4 m/s) for a total duration of 1 min in each instance. Lifting tasks were also tested and recorded. The lifting tasks included stooping (defined in this work as bending forward keeping the knees straight) and squatting (bending down while trying to keep the torso upright) alternately. The user started the motion from an upright position holding a 10 kg weight and performed the stoop or squat until the weight touched the ground. He then came back to the initial upright position. Two different modalities of motions were performed: (a) continuous flowing motion and (b) holding the lower (stoop/squat) position for 1 s.

#### 3.1.4 Evaluation of mechanical design

**Figure 4.**
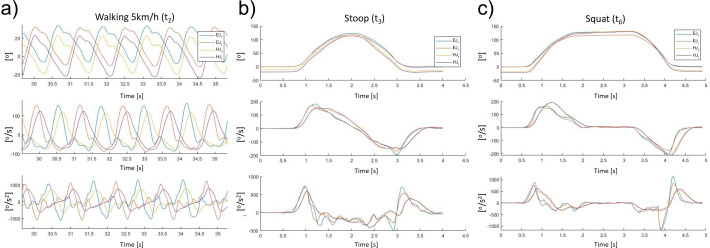
Joint angle variation ^o^, angular speed ^o^ s, and angular acceleration ^o^ s^2^ for right and left exoskeleton joints and user’s hips during following tasks: (a) walking at a constant speed of 5 km/hr, (b) stooping, starting from upright, and holding a 10 kg weight, and (c) squatting starting from upright, waiting for 1 s at full squat and then returning to upright, while holding a 10 kg weight.

In this section, a comparison is made between the human and exoskeleton kinematics. The human joint that was compared with the exoskeleton is the hip because, during the selected tasks, it has the most variation in terms of angular displacement. Results were recorded for mean absolute error, standard deviation and relative errors in angular displacement, speed, and acceleration, Table 1 The results presented in Table 1 show kinematic comparison data for the three tasks (i.e., walking, stooping, and squatting) presented in “Experimental test protocol” in the two modalities (continuous and intermittent). Figure 4a–c show angular displacement, speed, and acceleration during: walking at a constant speed of 5 km/hr, continuous stooping and squatting, respectively. *EJ_r_* and *EJ_l_* are the right and left exoskeleton joint profiles, while *HJ_r_* and *HJ_l_* represent the right and left human hip joint plots, respectively. From Figure 4a–c, it is evident that the profiles for the exoskeleton joints and hips in all three displayed tasks are different. Focusing, only, on the angular speed during these three tasks, the exoskeleton joint presents higher peak value compared with the user’s hip profiles. The average absolute error and standard deviation in the three displayed examples is 31.8^o^8 ± 43.9^o^ s (relative error of 25.3%) for the walking task (*t*_2_), 20.2^o^ ± 25.8^o^ s (relative error of 13.2%) for the continuous stooping task (*t*_3_), and 19 ± 29.4^o^ s (relative error of 9.1%) for the squatting task (*t*_6_). Similar consideration can be applied to the angular accelerations in all three tasks. The exoskeleton joint presents higher values compared with the user’s hip trends. The average absolute error and standard deviation in the three displayed examples are 314^o^ ± 412^o^ s^2^ (relative error of 40.6%) for the walking task (*t*_2_), 117^o^ ± 179^o^ s^2^ (relative error of 19.6%) for the stooping task (*t*_3_), and 151^o^ ± 235^o^ s^2^ (relative error of 26.1%) for the squatting task (*t*_6_).

**Table 1.** Mean absolute error (MAE), standard deviation (STD), and relative error (RE) for walking tests at 2.5 and 5 km/hr (*t*_1_, *t*_2_), stooping tests (*t*_3_, *t*_4_), and two modalities squatting tests (*t*_5_, *t*_6_).

Test	MAE	STD	RE
Angular displacement *t*_1_ (2.5 km/hr) [ ^o^]	8.7	1.4	39.2%
Angular displacement *t*_2_ (5 km/hr) [ ^o^]	12.6	1.3	47.8%
Angular displacement *t*_3_ (i) [ ^o^]	11.8	3.8	10%
Angular displacement *t*_4_ (ii) [ ^o^]	15.2	4.9	13.6%
Angular displacement *t*_5_ (i) [ ^o^]	9.9	7.5	7.2%
Angular displacement *t*_6_ (ii) [ ^o^]	8.2	7.6	6.2%
Angular speed *t*_1_ [ ^o^/s]	21.9	27.9	20.4%
Angular speed *t*_2_ [ ^o^/s]	31.8	43.9	25.3%
Angular speed *t*_3_ [ ^o^/s]	20.2	25.8	13.2%
Angular speed *t*_4_ [ ^o^/s]	13.4	18.9	8.4%
Angular speed *t*_5_ [ ^o^/s]	25.6	35.2	11.3%
Angular speed *t*_6_ [ ^o^/s]	19	29.4	9.1%
Angular acceleration *t*_1_ [ ^o^/s^2^]	191	245	32.8%
Angular acceleration *t*_2_ [ ^o^/s^2^]	314	412	40.6%
Angular acceleration *t*_3_ [ ^o^/s^2^]	117	179	19.6%
Angular acceleration *t*_4_ [ ^o^/s^2^]	93	143	20.1%
Angular acceleration *t*_5_ [ ^o^/s^2^]	157	235	22.7%
Angular acceleration *t*_6_ [ ^o^/s^2^]	151	235	26.1%

These results clearly show that although the kinematics of the human joint have a profile that approximately corresponds to those of the exoskeleton joint, for the exoskeleton joints, and the wearer’s hip, there are noticeable differences in angle, speed, and acceleration. Since the *XoTrunk* has been dimensioned based on traditional human kinematics analysis, the characterization of these differences may provide to a more accurate actuator sizing and proper control design. Indeed, as demonstrated, the traditional motion tracking approach led to a 19–40% relative error in angular acceleration, suggesting that more precise data gathered directly from the device kinematics are needed. From this analysis, when considering angular accelerations and speeds, the worst mismatch is generated during walking with deviation of as high as 48% relative error in angular position, 25% in speed, and 40% in acceleration. Furthermore, if the requirement is to replicate with the exoskeleton the human kinematic, this analysis will conduct to a redesign of the mechanical design of the exoskeleton kinematic chain to ensure more adherence with the human kinematics.

A secondary outcome of this experimental analysis is the full characterization of the exoskeleton joint in relation to the corresponding human joint. It is possible to characterize mathematically both kinematic systems (human and exoskeleton joints). Doing so, it is also possible to derive the human angular displacement as a function of the exoskeleton angular profile (more details are given in Appendix A1). This mathematical tool may enable a deep mechanical/ergonomic analysis.

### 3.2 Simulation of Actuation Performance

This step supports the selection of the actuator components and controllers by simulating the interaction of the wearer with the robotic system using an auxiliary disturbance motor that replicates human kinematic. The computer simulation makes use of the task-specific data obtained in the previous step (“Actuator Kinematics From Task-Specific Realistic Data”). In particular, the kinematic data from the walking task (*t*_2_, 5 km/hr) and lifting task (*t*_6_, continuous squat) have been selected for this implementation of the model-based simulation. These two tasks represent the worst-case scenario, in terms of kinematic variability, of both task categories: walking and lifting.

Unlike traditional test setups where dynamometers are used as the disturbance (Aghili et al., 2003; Di Natali et al., 2015), in this work, a disturbance motor is used to more completely and accurately replicate the speeds and accelerations that human kinematics undergo during the tasks. On the other side of the test setup, the exoskeleton actuator is connected to the output shaft of the disturbance motor, where it can apply a torque as if it were connected to the exoskeleton. An accurate plant model is the linchpin of control system development using model-based design. With a well-constructed plant model, the simulation allows the designer to verify the functionality of the control system, analyze the closed-loop model, and tune the gains. Moreover, the optimization of the hardware design, led by the model, supports the design procedure by identifying appropriate actuators for the requested task.

#### 3.2.1 Simulation setup description

**Figure 5.**
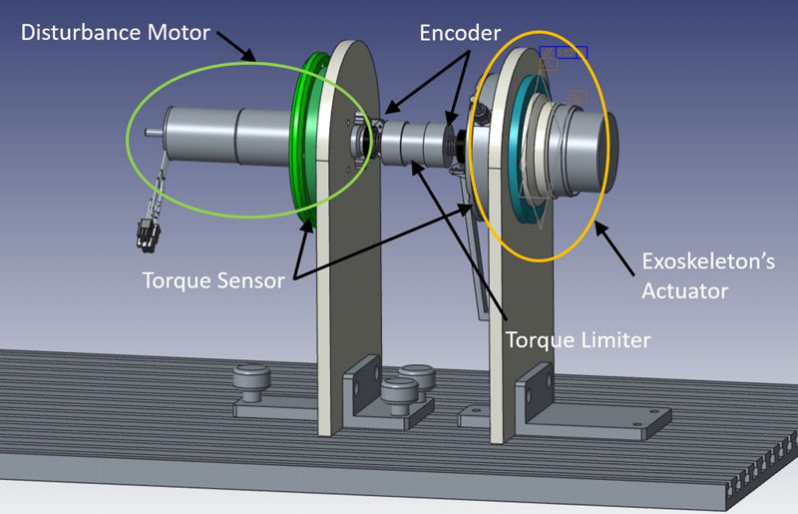
Rendering of the test setup. The disturbance motor is connected on the left of the torque limiter, with the exoskeleton actuator on the right.

Figure 5 illustrates the setup. The actuator, harmonic drive, and torque sensor (on the right of the torque limiter) replicate the setup on the exoskeleton (Toxiri et al., 2018a). The exoskeleton actuator is as described in “XoTrunk Exoskeleton Prototype.” On the left of the torque limiter is the disturbance motor. It is used to recreate the kinematics recorded in “Actuator Kinematics From Task-Specific Realistic Data” and thereby simulate the motion of the wearer while walking or bending over to accomplish a lifting task (i.e., stooping or squatting). The disturbance motor has been sized to be able to generate the needed kinematics in terms of speed and acceleration while subject to the assistive torque applied by the exoskeleton actuator on the right. The disturbance motor is an EC-i40, 100 W and 36 V supply (Maxon Motor AG, Switzerland) with a 1:26 planetary gear. For safety reasons, a torque limiter is used to connect the output shafts of the exoskeleton actuator and the disturbances actuator to prevent the transmission of impulsive torque beyond the mechanical limits. The torque limiter on the coupler plastic element also provides specific stiffness/damper values that are typical during human–machine interaction. Typical values of torsional damping and stiffness are in the range: *B_h_* = 10 Nms rad, *K_h_* = 50 Nm rad, respectively (Vette et al., 2014). Finally, several sensors are used to monitor the test workbench behavior and system states. In particular, a grounded torque sensor is positioned between the disturbance motor and the support, and two absolute encoders are located on opposite sides of the torque limiter to track the torque transmission and also close the control loop.

The *XoTrunk* exoskeleton implements a closed loop torque control based on torque sensor readings. During a lifting task, a possible assistance strategy is based on the trunk absolute orientation with respect to the ground (Toxiri et al. (2018a), Toxiri et al. (2018c)). Torque reference signals between 0 and 10 Nm are generated according to assistive strategy commonly used on the exoskeleton. Based on this strategy, the assistive torque increases in proportion to the increasing trunk inclination angles, with upright postures corresponding to no assistance. The torque reference signal (*T_D_*) has been generated by applying following function: *T_D_* = *T_M_* sin θ. Where *T_M_* is the maximum assistive torque value (10 Nm) and θ is the trunk inclination angle with respect to the ground. During the walking task, the user should not be aware of the exoskeleton’s inherent inertia, thus, the joint torque reference, measured by the exoskeleton, has to be kept null, that is, the torque reference signal is *T_D_* = 0 during the whole task execution. This following section addresses both tasks (walking and lifting) and gathers all the information needed to size the exoskeleton’s actuator. “Experimental Validation of Actuator Performance” and “Real-Scenario Evaluation of Actuation Performance” focus on showing how this approach improves system performance, and particularly, how it has been applied during the walking task to enhance transparency.

#### 3.2.2 Model and controller

**Figure 6.**
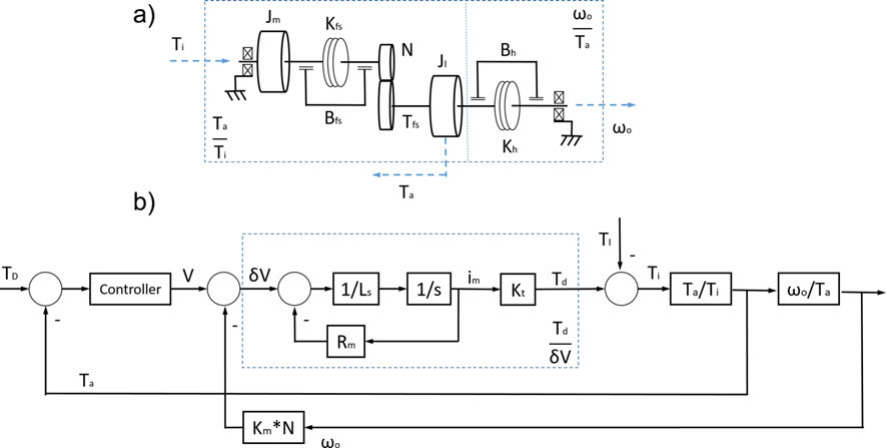
(a) Mechanical model of the exoskeleton’s actuator and a simplified human interaction model. (b) Block diagram of the whole system, including controller, electrical model, and mechanical model. Where *R_m_* is the motor resistance, *L_s_* motor inductance, *K_t_* motor torque constant, *K_m_* is the motor speed constant, and *N* is the transmission reduction.

The system model of the exoskeleton actuator is shown in Figure 6. Figure 6a represents the BLDC motor (Hai and Payandeh, 1997), the harmonic drive (Rabadi, 1995), and the human contact interaction (formed by combining damping and elastic values *B_h_* and *K_h_* as detailed in “Simulation setup description”). Figure 6b shows the block diagram of the closed loop torque control system for the electrical model (*T_d_* /δV), the physical model of the motor and harmonic drive (*T_a_* / *T_i_*), and the human interaction (ω*_o_* / *T_a_*). Where *T_d_* is the effective torque generated by the motor, δV is the voltage error input of the motor electrical model. *T_a_* is torque measured at the end of the actuation chain, and *T_l_* is the human torque disturbance due to the human–robot interaction. *T_i_* is the resultant torque after the human–robot interaction, while ω*_o_* is the angular speed measurement after the human interaction. The controller transforms the difference between the desired torque (*T_D_*) and measured torque (*T_a_*) into a voltage reference to drive the electrical motor model. The characteristic transfer function (ω*_o_* /V) of this system is:
(1)

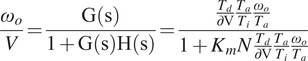

**Figure 7.**
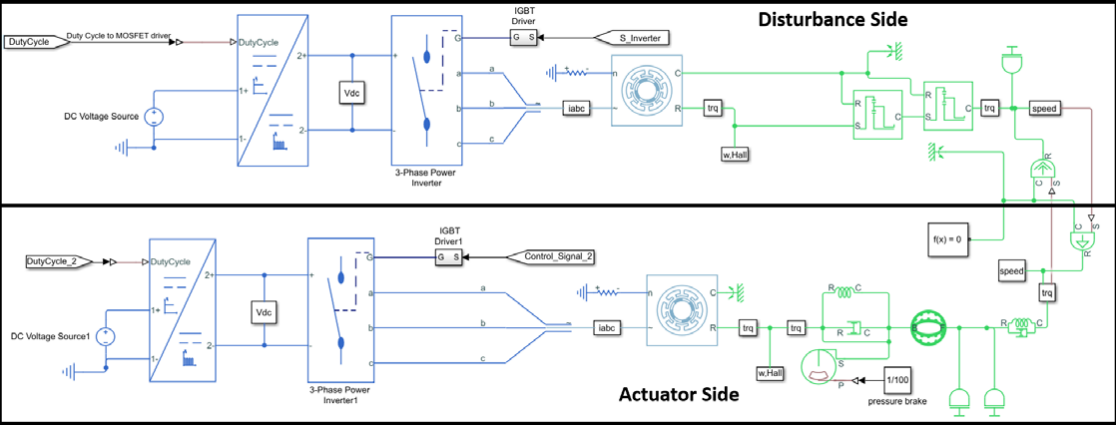
Electric and physical plants of disturbance motor and exoskeleton actuator. Both BLDC motors are driven by three-phase current and consequently, torque and speed are generated.

**Figure 8.**
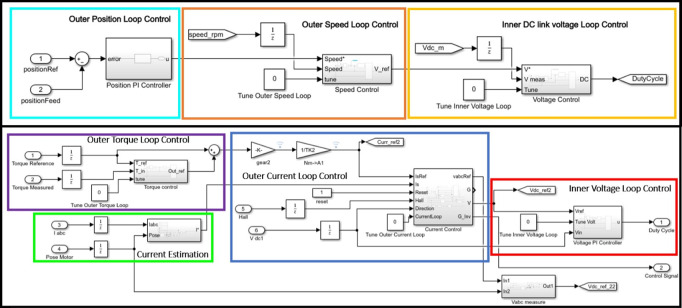
Disturbance and actuator side control loops.

The setup described above has been modeled in Simulink (MathWorks) using Simscape, and it is shown in Figure 7, including the electric and physical models (represented in blue and green, respectively) of the disturbance motor and the exoskeleton actuator. From the modeling point of view, the two systems are physically connected. In fact, the disturbance motor, that is used to replicate realistic kinematic data, recorded during the experimental tests of specific tasks (i.e., walking, stooping, squatting), is directly and physically connected to the exoskeleton actuator. The disturbance side then imposes a speed disturbance as a system input to the exoskeleton actuator, while the exoskeleton actuator imposes a torque input on the disturbance motor. From the electrical perspective, both motors are controlled by a duty cycle and control signal, which regulate the voltage supply and three-phase motor current through a four-quadrant chopper and an inverter. Figure 8 shows the control algorithms implemented for the disturbance motor and the exoskeleton actuator. The disturbance motor uses a PID-based position tracking control. The reference angular displacement has been recorded in the experimental trial presented in “Actuator Kinematics From Task-Specific Realistic Data.” The exoskeleton actuator control loop includes a forward PID torque tracker, a second closed loop PI current control with antiwindup, and a third stage of PI voltage regulation that generates the duty cycle signal. The current control loop relies on the current measurement being transformed from a three-phase motor current to a monophase equivalent current. This monophase current is obtained using the Park transform and then vector summing the two current components *i_q_* and *i_d_* (Chattopadhyay et al., 2011).

**Figure 9.**
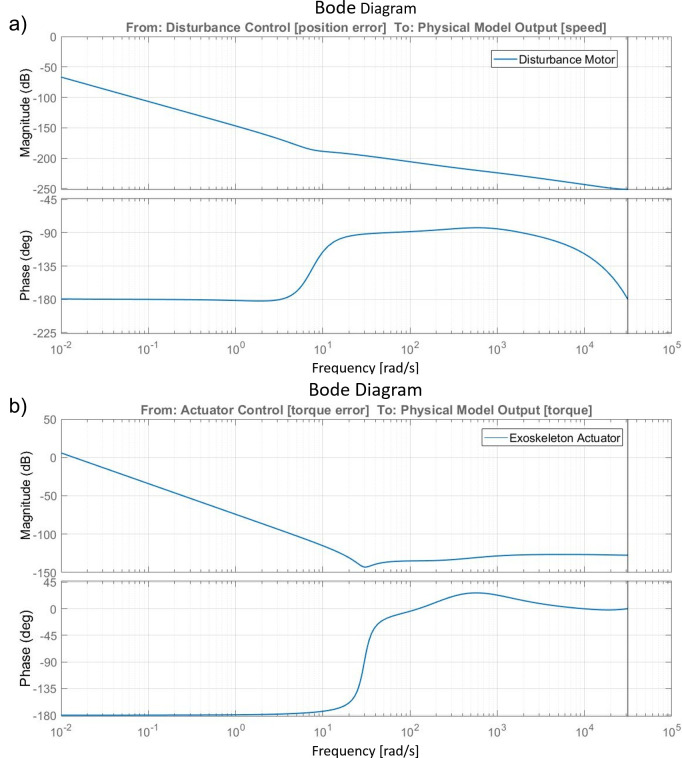
(a) Bode amplitude and phase charts of the disturbance motor system from the controller input (position error) to the physical model output (speed output). (b) Bode amplitude and phase charts of the exoskeleton actuator system from the controller input (torque error) to the physical model output (torque output).

The following analysis of the control and the simulation results shown in “Simulation and numerical results” are calculated based on the BLDC EC60 flat Maxon motor. The bode analysis represented in Figure 9 shows the open loop response of the disturbance side and the actuator side from the controller input to the system output. The controls coefficients, which have been selected taking advantage of the autotuner block function provided by Matlab/Simulink and then finalized with a fine manual tuning, are displayed in Table 2. The bode analysis underlines that both systems are stable in closed loop, none resonate, and antiresonant peaks in the transfer functions are present. For the disturbance side, the gain margin is 94 dB at 0.048 rad s, and the phase margin is 19^o^ at 0.00021 rad s. The actuator side results are asymptotically stable with infinite gain margin, while the phase margin is 0.008^o^ at 0.014 rad s.

**Table 2.** Control coefficients for disturbance and actuator side.

Controller	*K_p_*	*K_i_*	*K_d_*
Position	40.0	3.0	20.0
Speed	30.0	5.0	NA
Voltage (disturbance)	0.05	20.0	NA
Torque	20.0	200.0	0.8
Current	0.4	100.0	0.036
Voltage (actuator)	0.05	25.0	NA

*K_p_*, *K_i_*, and *K_d_* are proportional, integral, and derivative coefficients.

In the first instance, the simulation aims to select a suitable disturbance motor for the setup (see Appendix 6), as well as sizing the exoskeleton actuator. Moreover, the simulation enables design of the control strategies and parameter tuning on different tasks such as lifting and walking.

#### 3.2.3 Simulation and numerical results

**Figure 10.**
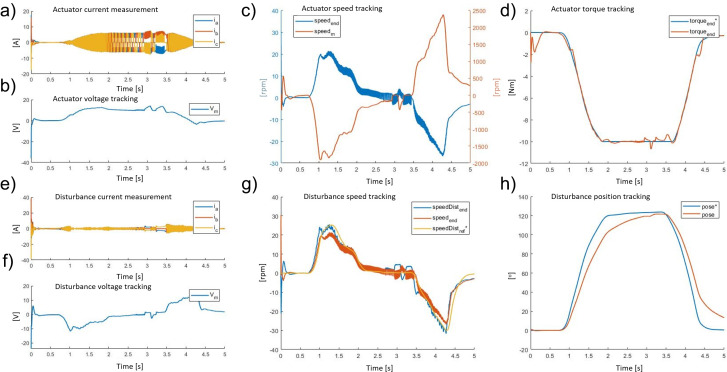
The exoskeleton actuator response during a walking task: (a) three-phase current, (b) voltage, (c) speed (motor side *speed_m_* and after the transmission *speed_end_*), and (d) torque measured (*torque_end_*) and its reference signal (*torque_e_* ^∗^*_nd_*). The disturbance motor response during a walking task: (e) three-phase current, (f) voltage, (g) speed (disturbance motor side *speedDist_end_*, actuator motor side *speed_end_*, and reference signal of disturbance motor side speed *speedDist_r_* ^∗^*_e f_*), and (h) position measured (*pose*) and reference signals (*pose* ^∗^).

The numerical results for the simulated kinematics on the BLDC EC60 flat Maxon while imposing torque assistance were as follows. Figure 10 shows both the actuator and disturbance side behavior in terms of current, voltage, speed, and position or torque during a lifting task while the disturbance motor simulates the task by applying kinematic and dynamic of the recorded data (“Actuator Kinematics From Task-Specific Realistic Data”). The signals with a ^∗^ represent the input reference signals for each of the physical quantities controlled in closed loop. The exoskeleton actuator control algorithm tracks the requested assistive torque to generate a torque error with a mean absolute error of 0.19 ± 0.36 Nm and a relative error of 1.9%.

**Figure 11.**
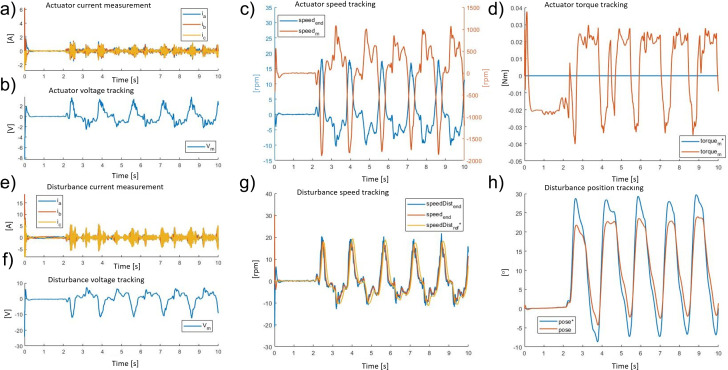
The exoskeleton actuator response during a walking task: (a) three-phase current, (b) voltage, (c) speed (motor side *speed_m_* and after the transmission *speed_end_*), and (d) torque measured (*torque_end_*) and its reference signal (*torque_e_* * *_nd_*). The disturbance motor response during a walking task: (e) three-phase current, (f) voltage, (g) speed (disturbance motor side *speedDist_end_*, actuator motor side *speed_end_*, and reference signal of disturbance motor side speed *speedDist_r_* * *_e f_*), and (h) position measured (*pose*) and reference signals (*pose* *).

Figure 11 shows both actuator and disturbance side behavior in terms of current, voltage, speed, and position or torque during the walking task, while the disturbance motor generates the recorded dynamics. The torque profile generated by the actuator oscillates alternately between plus and minus 0.02 Nm. This is due to the compensation for the system inertia and the fast dynamics changes needed to guarantee zero torque output at the end-effector (transparency). The exoskeleton actuator control algorithm tracks the requested null torque to guarantee a high level of transparency. The resultant torque mean absolute error is 0.29 ± 0.42 Nm and the relative error is 3%.

#### 3.2.4 Evaluation and consideration on the simulation

The model and simulation results provide an important input for the selection of the actuator components. For example, a particular sensor strategy leads to a hardware selection and to a coherent control design. Second, correct sizing of the actuator can be derived from the results that the simulation output provides.

For the exoskeleton actuator, from the analysis of the lifting task (Figure 10) and the walking task (Figure 11), it can be seen that the root mean square of the current consumption plot during maximum assistance is 3.1 A, and the maximum back EMF does not ever exceed 14.5 V. During the walking task, the root mean square of the current is approximately 2.2 A, and the maximum back EMF is always less than 12.5 V. As the exoskeleton motor (Maxon EC 60) has a voltage supply of 24 V and a nominal current of 5.6 A, it is clear that the maximum power usage by the system is about 33% of the total available power. The hypothesis that the exoskeleton’s actuator has been over-dimensioned has been proved, thus, a smaller motor could be selected without adversely affecting the overall motor performances but by reducing the weight the overall exoskeleton system performance, in terms of wearability, would be improved. By considering the maximum provided speed and torque, this analysis leads the selection of a motor with less power. Based on these considerations, the work reported in “Experimental Validation of Actuator Performance” and “Real-Scenario Evaluation of Actuation Performance” has been conducted with the exoskeleton’s actuator using the following BLDC motor model: Maxon EC 45 flat. (EC60-flat: nominal torque 0.23 Nm, stall torque 4.2 Nm, weight 0.47 kg; EC45-flat nominal torque 0.13 Nm, stall torque 1.5 Nm, weight 0.14 kg).

### 3.3 Experimental Validation of Actuator Performance

In this section, the validation on the physical test-bench (simulated in the previous step) is presented. The objective of this and the following section (“Real-Scenario Evaluation of Actuation Performance”) is to validate the transparency control mode. In particular, based on the data gathered in “Actuator Kinematics From Task-Specific Realistic Data,” it is clear that, for the controller, the walking tasks (*t*_1_ and *t*_2_ in Table 1) generate the worst scenario in terms of high kinematic variability. The assessment is conducted to compare several controllers and to determine which gives the best performance when subjected to a disturbance that replicates the user’s external motion. The authors propose four different tuned controllers to cover the uncertainty due to the human–robot interaction.

#### 3.3.1 Physical setup description

**Figure 12.**
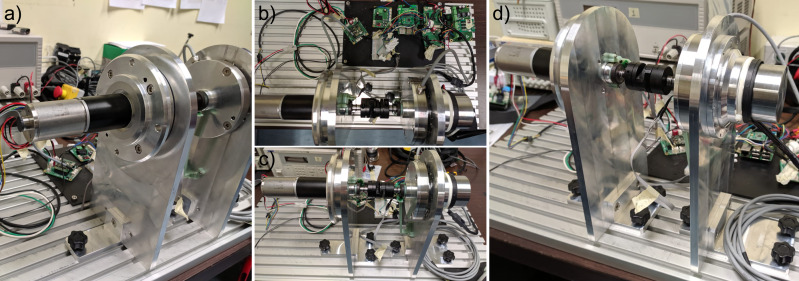
Pictures of the test workbench: (a) disturbance side, (b) electronics, (c) front view, and (d) actuator side.

**Figure 13.**
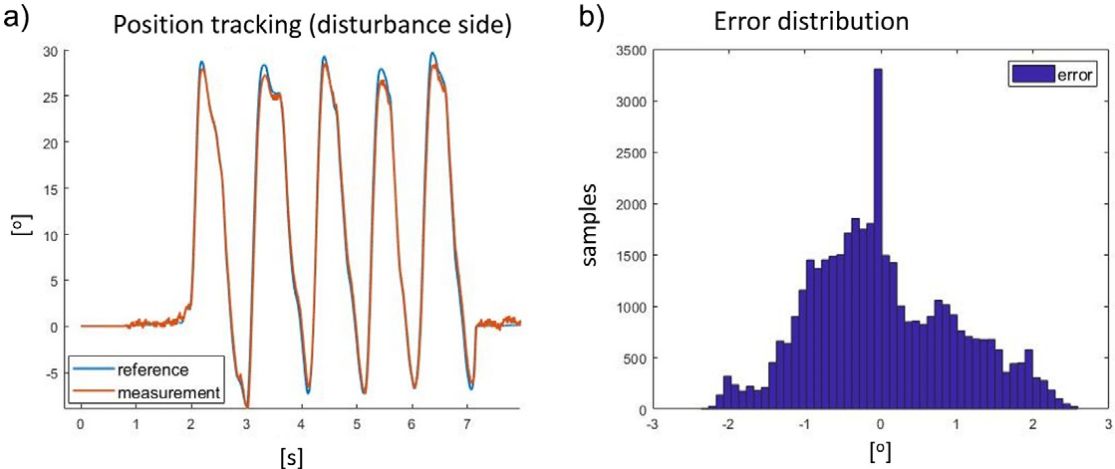
(a) Reference (blue) and result (red) of the pose tracking of the disturbance motor and (b) shows the distribution of tracking error expressed in degree.

The transparency mode is defined when the exoskeleton actuator follows the torque reference (selected at zero) while trying to generate no residual torque. Generated torque, in this configuration, is defined as the residual torque, and it adversely impacts the user’s comfort. Figure 12 shows the physical implementation of the test-bench described in “Simulation setup description.” The controller is shown in Figure 8. The parameters of the controllers in the real system are different from the simulated environment. This is because the current and voltage controllers are embedded into the BLDC motor drivers, and the details of the controller, such as PID parameters, are not available. Moreover, the motor used in the simulation and in the test-bench is not the same (simulation uses an EC60-flat, test-bench uses an EC45-flat). Therefore, the position and speed controllers of the disturbance motor have been retuned to reduce the tracking error. Finally, considering the disturbance side, the position and speed controllers use the coefficients presented in the first two lines of Table 3. The disturbance motor system shows good results with respect to the position tracking control performance. Figure 13 shows the actual tracking of the reference link position and the result using the proposed controller when walking at 5 km/hr. Numerically, the tracking performance results in an absolute average error and relative standard deviation of 0.75^o^ ± 0.94^o^, respectively.

**Table 3.** Control coefficients of the disturbance motor and the exoskeleton actuator.

Controller	*K_p_*	*K_i_*	*K_d_*	Low-pass freq
Position	35.25	2.5	15.75	NA
Speed	25.0	2.0	NA	NA
*Torque*_*p*1_	10	NA	NA	NA
*Torque*_*p*2_	20	NA	NA	NA
*Torque*_*p*3_	30	NA	NA	NA
*Torque*_1_	18	50.4	1.6	5 Hz
*Torque*_2_	18	223	0.36	10 Hz
*Torque*_3_	18	100	0.8	10 Hz
*Torque*_4_	18	36	2.0	5 Hz

*K_p_*, *K_i_*, and *K_d_* are proportional, integral, and derivative coefficients.

#### 3.3.2 Control design

The test-bench aimed to test and evaluate different possible PID controllers for the actuator side. These controllers and their coefficients are shown in Table 3. The first three controllers (*Torque*_*p*1_, *Torque*_*p*2_, *Torque*_*p*3_) are only proportional controllers, whereas the last four controllers are all PID (*Torque*_1_, *Torque*_2_, *Torque*_3_, *Torque*_4_). The proportional controller *Torque*_*p*1_ has been used previously with the current exoskeleton prototype and was selected to guarantee system stability (Toxiri et al., 2018a).

**Figure 14.**
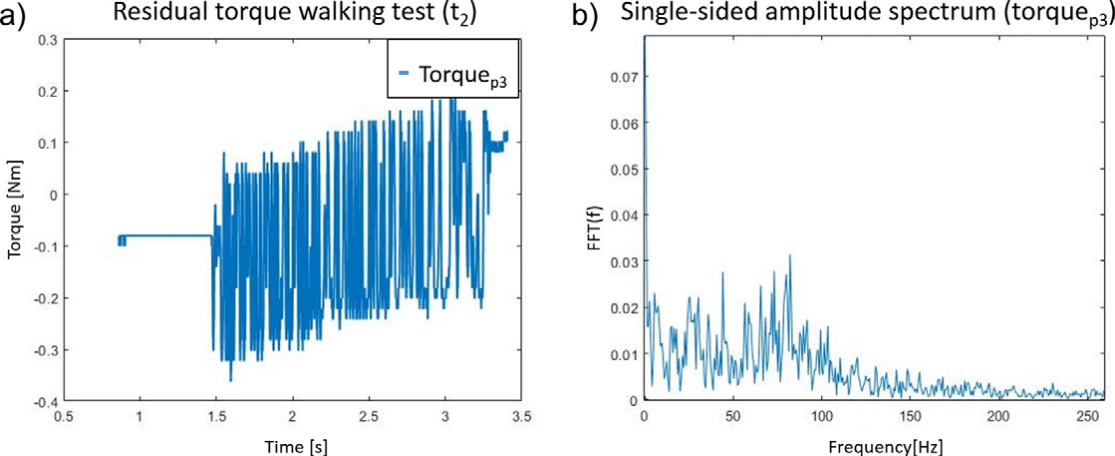
(a) Residual torque generated by the controller *Torque*_*p*3_, it shows oscillation in steady state and (b) fast Fourier Transform of the residual torque signal.

The controllers *Torque*_*p*2_ and *Torque*_*p*3_ have been tested to evaluate the effects of assuming more aggressive controllers. Both these controllers greatly increase the current demand, which results in an increasing probability of a BLCD driver fault condition. In particular, Figure 14a shows that the *Torque*_*p*3_ controller causes oscillations around the steady-state value of a constant amplitude. To tune the PID controllers, the Ziegler–Nichols method was used (Åström and Hägglund, 1995). The main frequency of oscillation (oscillation frequency [OF]) ranges from a few Hz to 50 Hz, as shown in Figure 14b. To choose a good trade-off between control responsiveness and controllability, we decided to evaluate four different controllers based on relative oscillation frequency spanning from 1 to 6.2 Hz. The frequency of motion of the human body is almost always below 10 Hz (Wall Iii et al., 2002; Zeng and Zhao, 2011). The PID controller, *Torque*_1_, has an OF 1.4 Hz, for *Torque*_2_ the OF is 6.2 Hz, with controller *Torque*_3_ the OF is 2.8 Hz, and finally, *Torque*_4_ has an OF of 1 Hz. The relative PID coefficients are shown in Table 3. To filter out any nose in the torque measurements fed to the controller, a low-pass filter at 5 or 10 Hz has been implemented. This filter prevents possible oscillation generated as the derivative of the noise. The selection of the low-pass filter cutoff frequency was predefined at 10 Hz. Where any residual oscillation at steady state was detected, the cutoff frequency was reduced at 5 Hz.

#### 3.3.3 Actuator performance evaluation

**Figure 15.**
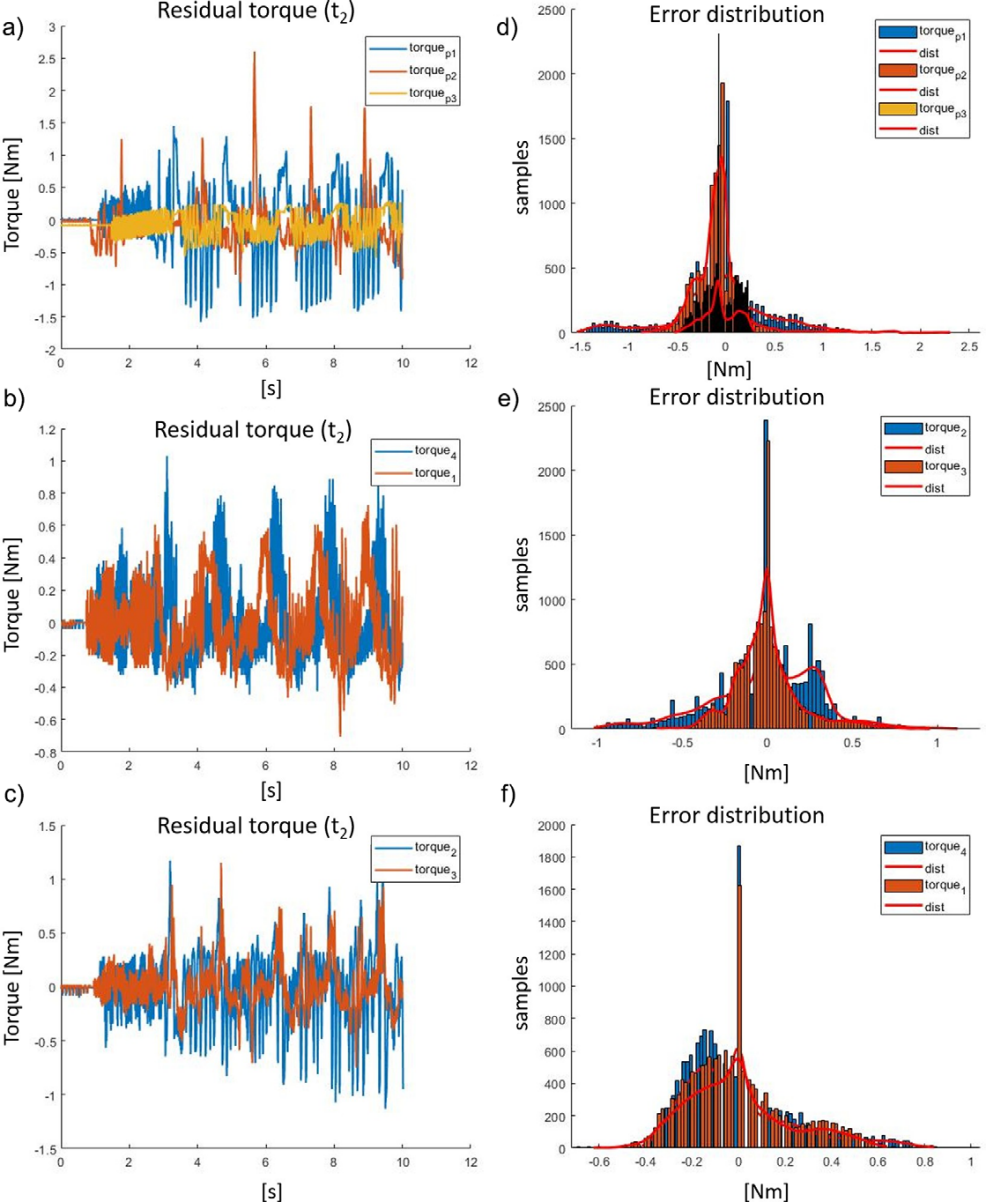
(a) Residual torque plots during a 10 s walking test (*t*_2_), of *Torque*_*p*1_, *Torque*_*p*2_, and *Torque*_*p*3_. (b) Residual torque plots during a 10 s test, of *Torque*_4_ and *Torque*_1_. (c) 10-second residual torque plots of *Torque*_2_ and *Torque*_3_. (d) Residual torque distribution comparison between *Torque*_*p*1_, *Torque*_*p*2_, and *Torque*_*p*3_. (e) Residual torque distribution of *Torque*_4_ and *Torque*_1_. (f) Residual torque distribution of *Torque*_2_ and *Torque*_3_. The controller parameters referred in this figure are in Table 4.

The assessment of the seven controllers (*Torque*_*p*1_, *Torque*_*p*2_, *Torque*_*p*3_, *Torque*_1_, *Torque*_2_, *Torque*_3_, and *Torque*_4_) involved using the disturbance motor to recreate the kinematic (angular position, speed, and acceleration) behavior of the test subject during the walking test. These data have been collected in “Actuator Kinematics From Task-Specific Realistic Data” and reported in Table 1. Each test replicate 10 s of walking pattern at 5 km/hr (test reference name *t*_2_). This corresponds to five steps. As the exoskeleton actuator control strategy (named transparency mode) aims to allow the user to freely move without experiencing any motion restriction, the torque reference has been set to zero for the duration of the test. During the test, the torque is recorded using the torque sensors on the bench-test (Figure 5) and the residual torque is calculated. The residual torque is defined as the torque error with respect to the reference torque, which can oscillate above and below the target value (torque reference is zero for the specific test *t*_2_). Testing and results of the reproduced kinematics on the exoskeleton actuator (embedding Maxon EC 45) and imposed torque reference (transparency mode, torque set at zero) are shown in Figure 15. The performance of the studied controllers has been evaluated by comparing the residual torque distributions as represented in Figure 15d–f. In addition, the percentiles of the residual torque are presented in Table 4. The overall performances of each controller is evaluated with respect to *Torque*_*p*1_. The analysis has been quantified by comparing the distribution of the residual torque between the 1st and 99th percentiles and the 25th and 75th percentiles. Table 5 shows the abovementioned comparison; *Torque*_1_, *Torque*_3,_ and *Torque*_4_ perform best in the test-bench, whereas *Torque*_*p*3_ is unstable. In particular, *Torque*_3_ reduces the residual torque by about 70% with respect to *Torque*_*p*1_ in the 25th to 75th range (the residual torque is reduced 3.5 times). *Torque*_1_ reduces the residual torque by approximately 60% within the 1st−99th range (it is reduced 2.6 times). The above results while being promising are still only tests in isolation on a “benchtop,” and it is important to develop this testing further through real human interaction trials involving experimental evaluation in the real scenarios. This will be presented in “Real-Scenario Evaluation of Actuation Performance.”

**Table 4.** Control performances for actuator side (data expressed in Nm).

Controller	1%	25%	50%	75%	99%
*Torque*_*p*1_	−1.39	−0.32	0.0	0.24	1.15
*Torque*_*p*2_	−0.65	−0.20	−0.08	0.0	1.45
*Torque*_*p*3_	−0.44	−0.18	−0.08	0.08	0.24
*Torque*_1_ lpf 5 Hz	−0.42	−0.16	−0.02	0.10	0.54
*Torque*_2_ lpf 10 Hz	−0.89	−0.18	0.0	0.22	0.79
*Torque*_3_ lpf 10 Hz	−0.42	−0.10	0.0	0.06	0.67
*Torque*_4_ lpf 5 Hz	−0.36	−0.16	−0.04	0.10	0.71

**Table 5.** Control performances for the actuator side in terms of reduction ratio between the 1st to 99th percentiles and between 25th and 75th percentiles with respect to *Torque*_*p*1_ performance (data expressed in %).

Controller	Range reduction 1–99%	Range reduction 25–75%
*Torque*_*p*2_	17.75	64.29
*Torque*_*p*3_	73.03	53.57
*Torque*_1_ lpf 5 Hz	61.93	53.57
*Torque*_2_ lpf 5 Hz	34.16	28.57
*Torque*_3_ lpf 5 Hz	57.17	71.43
*Torque*_4_ lpf 5 Hz	58.29	53.57

**Table 6.** Control coefficients for disturbance and actuator side.

Controller	*K_p_*	*K_i_*	*K_d_*	Low-pass freq
*Torque*_*p*1_	10	NA	NA	NA
*Torque*_1_	18	50.4	1.6	1 Hz
*Torque*_2_	18	223	0.36	5 Hz
*Torque*_3_	18	100	0.8	3–5 Hz
*Torque*_4_	18	36	2.0	1 Hz

*K_p_*, *K_i_*, and *K_d_* are proportional, integral, and derivative coefficients.

### 3.4 Real-Scenario Evaluation of Actuation Performance

In this section, the low-level controllers presented and developed within the previous sections (“Simulation of Actuation Performance” and “Experimental Validation of Actuator Performance”) have been implemented and assessed in a real scenario. The previous actuator configuration of the prototype A, described in “XoTrunk Exoskeleton Prototype,” has now been updated based on the findings of “Simulation of Actuation Performance,” with the EC60 motor being replaced by an EC45. This section aims to compare the performance of the controllers developed in “Experimental Validation of Actuator Performance” and also compare the best controller against the exoskeleton configuration (prototype A) described in “XoTrunk Exoskeleton Prototype.”

#### 3.4.1 Experimental trial

The experimental validation has been carried out on the walking task on a treadmill at 5 km/hr speed for 30 s. The tests used the smaller version of the motors (Maxon EC 45 flat) because it has been identified as more suitable for the task “Evaluation and consideration on the simulation.”

Then, the performance of the best controller was compared with the prototype A of the exoskeleton (motor EC 60 and controller *Torque*_*p*1_). Since the exoskeleton behaves differently from the bench-test system, due to the different front-end sensors noise, the low-pass filters were set in the range 1–5 Hz, instead of the 5–10 Hz range used in the bench-test evaluation (low-pass filters are displayed in Table 5). The torque reference was set to be null, as required in the transparent mode. The test was repeated three times for each of the five different controllers (*Torque*_*p*1_, *Torque*_1_, *Torque*_2_, *Torque*_3_, and *Torque*_4_). Each of the PID controllers (*Torque*_1_, *Torque*_2_, *Torque*_3_, and *Torque*_4_) has been compared against the proportional controller *Torque*_*p*1_. The test sequences on the *Torque*_3_ controller have been taken with two different low-pass filers: 3 and 5 Hz. The numerical results have been averaged over the three trials.

#### 3.4.2 Experimental evaluation

**Figure 16.**
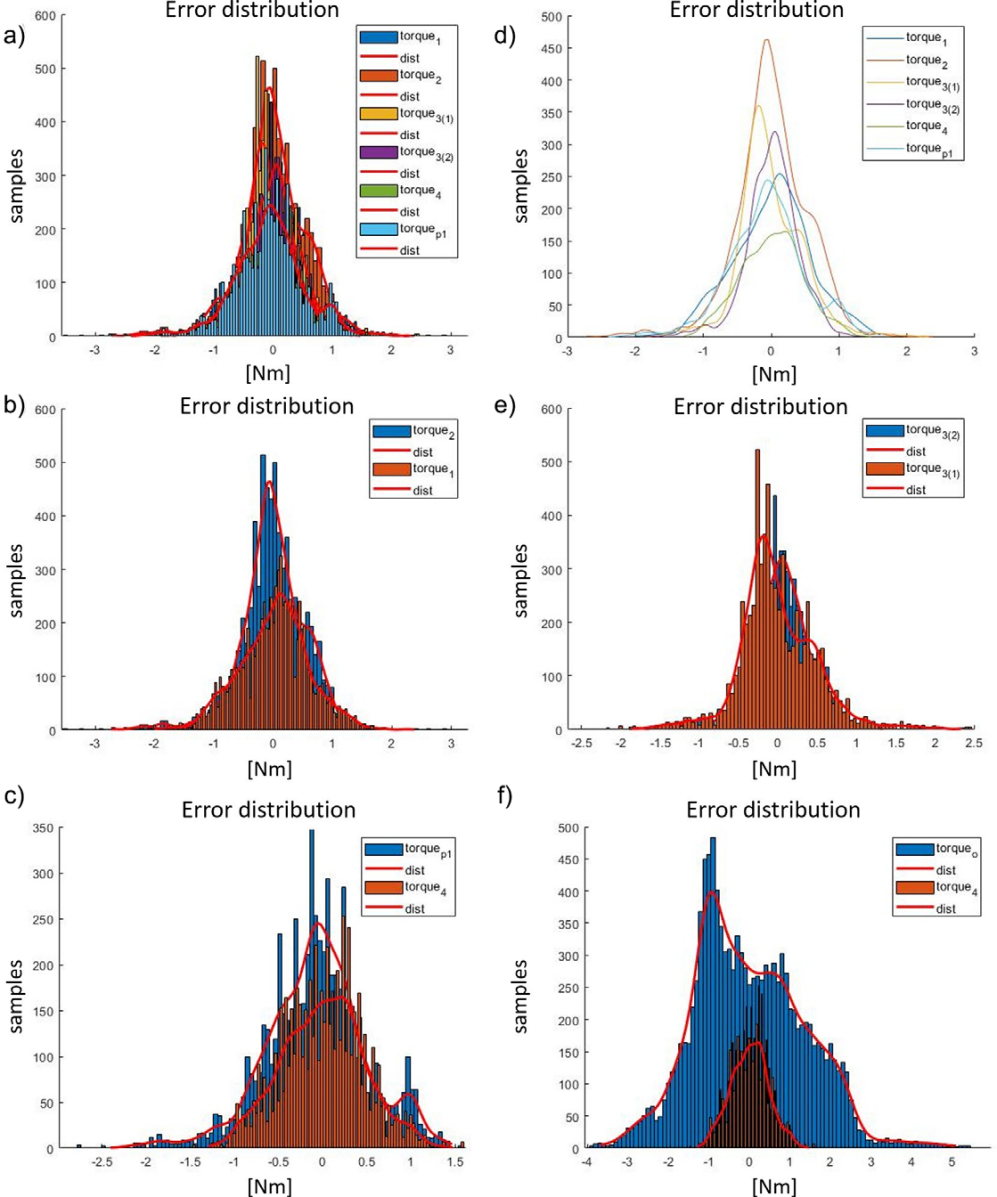
(a) Residual torque distribution (displayed with histogram) during walking test (*t*_2_) of all the evaluated controllers presented in Table 6. (b) Residual torque distribution comparison between *Torque*_2_ and *Torque*_1_. (c) Residual torque distribution comparison between *Torque*_*p*1_ and *Torque*_4_. (d) Residual torque distribution of all the evaluated controllers. (e) Residual torque distribution comparison between *Torque*_3 2_ (lpf 5 Hz) and *Torque*_3 1_ (lpf 3 Hz). (f) Residual torque distribution comparison between *Torque_o_* (actuator of the old exoskeleton’s version) and *Torque*_4_.

The description of the torque performance and numerical results of the walking task in transparent mode is displayed in Figure 16. The percentiles of the residual torque are presented in Table 7. The overall performance of the controllers is evaluated against the *Torque*_*p*1_ controller within the 1st to 99th and the 25th to 75th ranges. The results are shown in Table 8. The *Torque*_3_ (5 Hz low-pass filter) controller performs well, although there is some vibration due to noise. It reduced the residual torque in the range 25th to 75th to about 35%. The *Torque*_4_ performs the best in the real scenario, reducing the full range of residual torque to about 30%. In conclusion, the *Torque*_4_ controller with low-pass filter at 1 Hz is not affected by vibration due to residual noise, and it performs better than all the other controllers.

**Table 7.** Control performances for actuator side expressed in Nm.

Controller	1%	25%	50%	75%	99%
*Torque*_*p*1_	−1.81	−0.42	−0.06	0.26	1.21
*Torque*_1_ lpf 1 Hz	−1.37	−0.36	0.06	0.38	1.33
*Torque*_2_ lpf 5 Hz	−1.9	−0.28	0.0	0.34	1.23
*Torque*_3_ lpf 3 Hz	−1.33	−0.28	−0.06	0.3	1.56
*Torque*_3_ lpf 5 Hz	−1.27	−0.2	0.02	0.24	0.91
*Torque*_4_ lpf 1 Hz	−1.02	−0.32	0.02	0.3	1.09

**Table 8.** Control performances related to the controller *Torque*_*p*1_ and expressed in %.

Controller	Range reduction 1–99%	Range reduction 25–75%
*Torque*_1_ lpf 1 Hz	10.03	−8.8
*Torque*_2_ lpf 5 Hz	−3.68	8.8
*Torque*_3_ lpf 3 Hz	4.35	14.7
*Torque*_3_ lpf 5 Hz	27.33	35.3
*Torque*_4_ lpf 1 Hz	30.1	8.8

Finally, the *Torque*_4_ controller has been, also, compared against the prototype A of the exoskeleton (description provided in “XoTrunk Exoskeleton Prototype”). Table 9 shows distribution percentiles of both the *Torque*_*p*1_ and the *Torque*_4_ controllers, and Table 10 shows the comparison of performance and torque range reduction. The result shows a reduction of the maximum absolute residual torque in a zero-tracking torque control mode of over three times if considering the percentile range 25th to 75th (from 1.92 to 0.62 Nm). The system improvements are due to basing the actuator sizing on the kinematics analysis derived from computer simulations and the test-bench experiments, and this has directly led to improved actuator performance and design and selection of more suitable controllers.

**Table 9.** Control performances for actuator side expressed in Nm.

Controller	1%	25%	50%	75%	99%
*Torque*_4_ lpf 1 Hz *Torque*_*p*1_ original version	−1.02 −2.79	−0.32 −0.96	0.02 −0.13	0.3 0.96	1.09 2.54

**Table 10.** Control performances related to the controller *Torque*_*p*1_ applied on the original exoskeleton version and expressed in %.

Controller	Range reduction 1–99%	Range reduction 25–75%
*Torque*_4_ lpf 1 Hz	60.4	67.4

## 4 Conclusions

The complexity of human–robot interaction particularly while wearing an exoskeleton means that safe, smooth, accurate, predictable motions, and high user comfort are paramount. Under such conditions, approximate controllers or poorly dimensioned actuators that would generate low performance and uncomfortable effects are not tolerable. To address these critical design issues, this paper explored a learning-based evaluation framework, taking into consideration the human–robot interaction, to support the design and analysis of mechanical, actuation, and control solutions. This was illustrated using an industrial back-support exoskeleton as the case study, but the framework and physical setup will generalize very well to different combinations of exoskeleton, collaborative robots, and physical tasks. The study is based on an analysis of human–robot interaction, by modeling the human joint as a position input, the exoskeleton as torque source, and considering their interaction as a combination of elastic and damping physical components (see “Simulation setup description”). This selection of components and parameters is driven by two concerns: the desired behavior during a series of real-world-inspired tasks and the nature of the assistive wearable device that we are studying. This work demonstrates the development and use of an iterative methodology to improve exoskeleton design and development phases while supporting engineering choices with data gathered from real tasks.

As a first finding, the work underlines the differences in terms of kinematics between an exoskeleton and the corresponding human skeleton and how this impacts the actuator design/selection. This kinematic mismatch should not be considered as a disadvantage but, rather, should be evaluated and quantified as a system design feature. The problem is initially approached by recording a series of case study kinematics (i.e., walking and lifting tasks) which generate position, speed, and acceleration profiles. The gathered data of the exoskeleton joint kinematics and the subsequent comparison against associated human joint (i.e., hips) underlined the hypothesis that there was a significant mismatch. This mismatch (i.e., the worst-case scenario) was generated during walking tasks with a 48 and 40% relative error in the angular position and acceleration, respectively. This worst-case (high kinematic variability) scenario was subsequently replicated on a physical test-bench, to facilitate accurate control optimization and evaluation of the actuator performance. A second important result from the computer simulations and test-bench experiments was a new approach to the selection of more suitable actuator components and on the analysis of controller performance. From the case studies, this work identified that it was often possible to select a lower-powered motor than conventional design approaches suggested. This use of smaller motors improves a number of aspects including vitally, the user experience and overall weight. Experimental validation on a test-bench enabled the controllers to be fine-tuned, and performance comparison to be made which were then easily transferred directly onto the exoskeleton system. Finally, the real-scenario evaluation extends the previous steps by considering the human interaction. Testing of the framework on the test exoskeleton and during the physical tasks showed that the performance was improved by between 300 and 400% compared to the initial configuration.

As with all systems, the work presented in this paper has a number of limitations that should be acknowledged, and these will be explored in future work. The very small sample size (in both numbers of subjects and scenarios) for this study limits the generalizability of the data, although we believe the framework extends naturally to bigger datasets. Interaction forces between the exoskeleton and the user are empirically observed to cause the device to move. This behavior could affect the recorded dynamics, thus further investigation by comparing also the prototype A of the exoskeleton would be valuable. Future work will also consider using the approach to test and validate many more test scenarios including lifting tasks, stepping up and down, and lunging. Finally, consideration of the physical human interaction, beyond simply the elastic coupling and damping effect, will be studied to improve the modeling and the analysis of the controller.

## Figures and Tables

**Figure 17.**
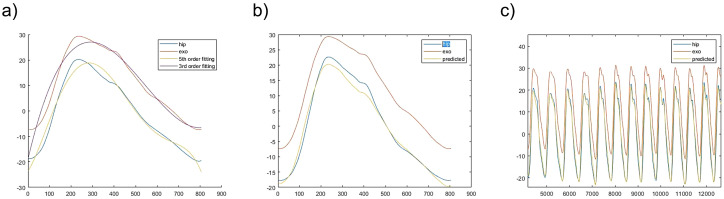
(a) Fittings of exoskeleton joint angle (third-order polynomial fitting) and hip angle (fifth-order polynomial fitting). (b) The figure shows the estimated trend as function of the exoskeleton angular trend. (c) The estimated trend after multiple walking cycles.

## Appendix A1

In this section, the implementation of a mathematical tool to achieve the hip joint angle as a function of the exoskeleton joint trend is presented. The proposed method is the inverse of composite polynomial functions. By deriving this relationship, it would provide the prediction of the human joint as a function of the exoskeleton joint trend. Because of the analytic expression, this mathematical tool may enable more deep mechanical/ergonomic analysis and design, as well as, it enables for strategy of sensor-free monitoring of human joint behavior.

Consider the polynomial fittings for the synchronized time-variant plot of the exoskeleton joint angle and hip joint angle. The hip joint angle can be expressed as a function of the exoskeleton joint angle as demonstrated in the following steps. The exoskeleton joint is given by:

The hip angle is:

(2)

(3)

As any hip angular displacement would cause a change on the exoskeleton joint angle. This relationship may be considered as black box (**j** *x*), which gets as its input is the exoskeleton’s joint angle (**f**(t)), while the system output is the hip angle (**g**(t)). The composite function is as following:

(4)

If we impose the Equation (3) being equal to the Equation (4), the composite function can be expressed as following:

(5)

Considering that the inverse function of Equation (2) (**f** ^−1^ *t*) returns the time as function of the exoskeleton’s angle as: *t* θ*_ex o_.* We can substitute the time (*t*) of the Equation (3) with the inverse of equation 2 as following:

(6)

The application of Equation (6) returns the estimation of the hip angle as function of the exoskeleton joint angle. Figure 17a shows both polynomial fitting of exoskeleton joint and hip angles. Figure 17b shows the estimation of the hip angle as a function of the inverse of the exoskeleton’s joint angle in a single walking gate. Finally, Figure 17c shows an extract of the walking experiment, in which the hip angle estimation is overlaid to the measured hip angle. The prediction mean absolute error with respect to the hip angle direct measurement, is 2 ± 2.4 degrees. The relative error is the 4% of the maximum range of angular variation. Considering these results, this method can be used to enable a strategy for sensor-free monitoring of human joint.

## Appendix A2

This section shows the dimensioning of the disturbance motor. The disturbance motor trends during lifting task are presented in Figure 10. In particular, Figure 10e shows the three-phase current, Figure 10f shows the back EMF, Figure 10g shows the end-effector side reference and measured speed, Figure 10h shows the position reference and output tracking of the disturbance motor, while assistance torque was applied by the exoskeleton actuator. The control algorithm of the disturbance side performs a position tracking with a mean absolute error of 10.6  ± 13.6^o^ and relative error 8.6%.

The disturbance motor trends during walking task are presented in Figure 10. In particular, Figure 11e shows the three-phase current, Figure 11f shows the back EMF, Figure 11g shows the speed at the end of the transmission train, and Figure 11h shows the position reference and tracking of the disturbance motor. The control algorithm of the disturbance side performs a position tracking with a mean absolute error of 2.96 ± 3.68^o^ and relative error 9.9%.

In the case study of the lifting task, for the particular disturbance motor (Figure 10), the root mean square of the current values and maximum voltage are about 2.08 A and 14.6 V, respectively, whereas during walking task (Figure 11), the root mean square of the current is about 0.48 A and the maximum of the supplied voltage is less than 4.3 V. Therefore, the disturbance motor is well dimensioned. For the specific motor, there is not necessity of maximizing performance, but rather the need to ensure only a confidence margin of performance.
